# Understanding the neurological implications of acute and long COVID using brain organoids

**DOI:** 10.1242/dmm.050049

**Published:** 2023-07-17

**Authors:** Laura García-González, Andrea Martí-Sarrias, Maria C. Puertas, Ángel Bayón-Gil, Patricia Resa-Infante, Javier Martinez-Picado, Arcadi Navarro, Sandra Acosta

**Affiliations:** ^1^ Institute of Neurosciences, Department of Pathology and Experimental Therapeutics, Medical School, Universitat de Barcelona, L'Hospitalet de Llobregat 08907, Spain; ^2^ Barcelonaβeta Brain Research Center, Pasqual Maragall Foundation, Barcelona 08005, Spain; ^3^ Institute of Evolutionary Biology (UPF-CSIC), Department of Medicine and Life Sciences, Universitat Pompeu Fabra, Barcelona 08003, Spain; ^4^ IrsiCaixa AIDS Research Institute, Badalona 08916, Spain; ^5^ CIBERINFEC, Instituto de Salud Carlos III, Madrid 28029, Spain; ^6^ Germans Trias i Pujol Research Institute (IGTP), Badalona 08916, Spain; ^7^ University of Vic-Central University of Catalonia (UVic-UCC), Vic 08500, Spain; ^8^ Catalan Institution for Research and Advanced Studies (ICREA), Barcelona 08010, Spain; ^9^ Center for Genomic Regulation (CRG), The Barcelona Institute of Science and Technology, Barcelona 08003, Spain; ^10^ Neurodevelopmental Disorders Lab, Neuroscience Program, Institut d'Investigació Biomèdica de Bellvitge, IDIBELL, L'Hospitalet de Llobregat 08908, Spain

**Keywords:** SARS-CoV-2, Long COVID, Neurological affectations, Brain organoids

## Abstract

As early as in the acute phase of the coronavirus disease 2019 (COVID-19) pandemic, the research community voiced concerns about the long-term implications of infection. Severe acute respiratory syndrome coronavirus 2 (SARS-CoV-2), like many other viruses, can trigger chronic disorders that last months or even years. Long COVID, the chronic and persistent disorder lasting more than 12 weeks after the primary infection with SARS-CoV-2, involves a variable number of neurological manifestations, ranging from mild to severe and even fatal. *In vitro* and *in vivo* modeling suggest that SARS-CoV-2 infection drives changes within neurons, glia and the brain vasculature. In this Review, we summarize the current understanding of the neuropathology of acute and long COVID, with particular emphasis on the knowledge derived from brain organoid models. We highlight the advantages and main limitations of brain organoids, leveraging their human-derived origin, their similarity in cellular and tissue architecture to human tissues, and their potential to decipher the pathophysiology of long COVID.

## Teaser

**Summary:** Brain organoids recapitulate human brain cytoarchitecture *in vitro*, and are likely to become key models to disentangle the neuropathology of acute and long COVID, and other potential long-term sequelae of COVID-19.

## Introduction

The emergence and rapid spread of severe acute respiratory syndrome coronavirus 2 (SARS-CoV-2), as well as its variable pathogenicity, have jeopardized global health, infecting at least 767 million people worldwide and killing more than 6.9 million as of June 2023 [World Health Organization (WHO) epidemiological update, 15 June 2023]. During this time, we have witnessed and been part of a worldwide effort to coordinate and advance research and clinical solutions that resulted in fast implementation of comprehensive vaccination plans, as well as in several treatments and preventative actions to reduce the impact of coronavirus disease 2019 (COVID-19). The coordination of the scientific community has been boosted by the application of emerging technologies for the study of infectious diseases, such as advanced *in vitro* cell cultures and artificial intelligence (AI) algorithms. Three-dimensional (3D) cell cultures, and especially organoids, have helped researchers to understand the acute cellular pathophysiology of SARS-CoV-2 (Lamers et al., 2020, 2021a; Monteil et al., 2020; Ramani et al., 2020; Song et al., 2021a). Other technologies, such as AI algorithms or RNA-based vaccines, have shed light on several aspects of COVID-19 pathophysiology and outcome risks of the population immunization (Borkowski et al., 2020; Dite et al., 2021; Goncharov et al., 2021). This high volume of resources has drastically reduced fatalities, especially in highly medicalized countries. However, long-lasting sequelae remain a major health burden for a significant proportion of the SARS-CoV-2-infected population, including the persistence and onset of novel symptoms. This is commonly known as long COVID or as ‘post-acute sequelae of COVID-19’.

**Fig. 1.**
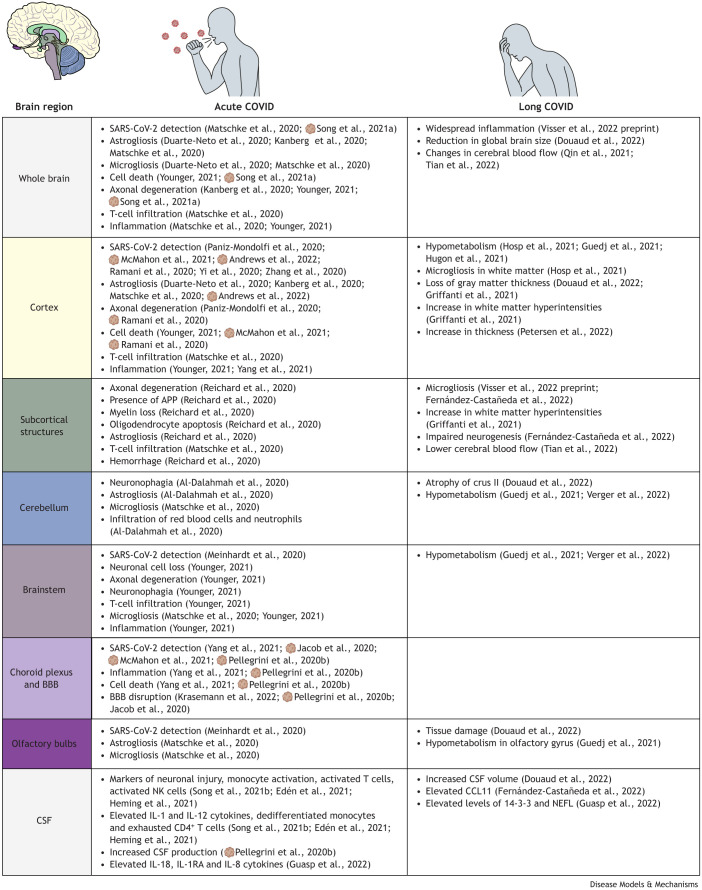
**Effects of acute and long COVID in the CNS.** The phenotypes that have also been observed in brain organoids after short-term *in vitro* infections are marked with organoid icons. BBB, blood–brain barrier; CNS, central nervous system; COVID, coronavirus disease; CSF, cerebrospinal fluid; NK, natural killer; SARS-CoV-2, severe acute respiratory syndrome coronavirus 2.

Many questions remain unanswered about the pathophysiology of long COVID, including its causes, potential genetic risks or how comorbidities influence its evolution. In this Review, we summarize the pathological events in the central nervous system (CNS) upon SARS-CoV-2 infection (Fig. 1), and how these relate to the development and persistence of long COVID. Furthermore, we survey the current experimental approaches to explore these mechanisms, focusing on how advanced 3D tissue cultures can help to understand the pathogenesis of (long) COVID in the CNS and on their potential to broaden a new therapeutic horizon.

**Fig. 2.**
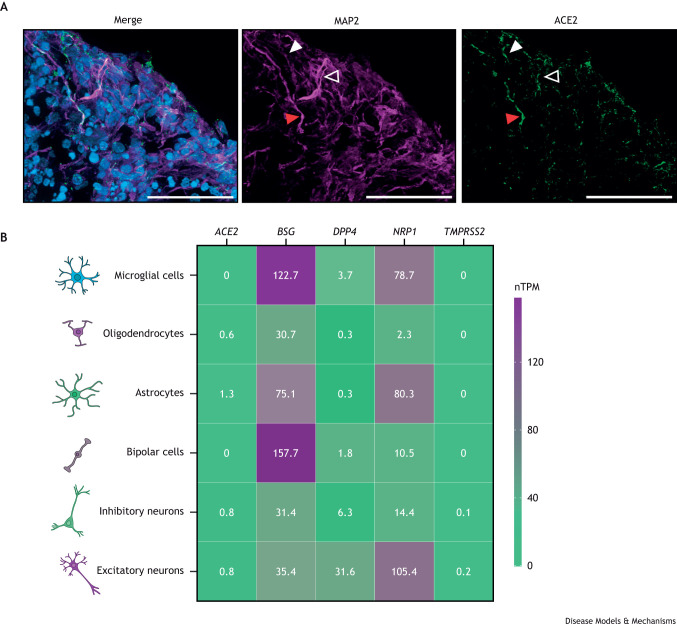
**Host factors, receptors and other proteins involved in SARS-CoV-2 infection in the brain.** (A) Neurons express the ACE2 receptor, permitting entry of SARS-CoV-2. Immunofluorescent staining of 180-day-old brain organoids for ACE2 (green) and the neuron-specific cytoskeletal marker MAP2 (magenta). Nuclei are stained with 4′,6-diamidino-2-phenylindole (DAPI; blue). Open arrowhead shows a neuron (MAP2^+^) with no ACE2 expression; white-filled arrowhead shows a non-neuronal cell (MAP2^−^) with ACE2 expression; red-filled arrowhead shows a neuron (MAP2^+^) expressing ACE2. Scale bars: 50 μm. This image was generated in the S.A. laboratory by A.M.-S. and Isabel Turpín. (B) A number of brain cell types express genes for which the products facilitate SARS-CoV-2 infection. The heatmap shows expression levels of *ACE2*, *BSG* (*CD147*), *DPP4*, *NRP1* and *TMPRSS2* mRNA in brain cell subtypes according to single-cell RNA-sequencing data from the Human Protein Atlas. Expression levels are expressed in normalized transcripts per million (nTPM).

SARS-CoV-2 infection occurs when the virus spike (S) protein binds to the zinc metalloproteinase angiotensin-converting enzyme 2 (ACE2) at the host cell membrane (Hoffmann et al., 2020a; Lan et al., 2020; Shang et al., 2020a; Wan et al., 2020) (Fig. 2). *In vivo*, ACE2 expression is low in most neural cell types; however, it is expressed strongly in other cell types, such as cardiomyocytes. Subsequently, three different cellular internalization processes have been described:

(1) Clathrin-mediated endocytosis internalizes the ACE2-bound viral particles. Once inside the endosomes, the acidic pH facilitates cellular cathepsin L-mediated priming of viral S protein, resulting in endosomal membrane fusion with the viral envelope (Bayati et al., 2021; Ou et al., 2020).
(2) Alternatively, in the non-endosomal pathway, the primary activation is based on the cleavage of S protein by the cellular transmembrane serine protease 2 (TMPRSS2) (Koch et al., 2021) and subsequent spike cleavage by the endonuclease furin (Bestle et al., 2020; Shang et al., 2020b). These proteases cleave the ACE2-bound viral S protein, forming a membrane pore as a result of fusion of the cell membrane and viral envelope, with subsequent release of viral RNA into the cytoplasm (Hoffmann et al., 2020b). Moreover, the direct interaction between ACE2 and TMPRSS2 enhances S-driven membrane fusion despite its cleavage (Essalmani et al., 2022).
(3) Viral particles bound to soluble ACE2, which is cleaved by the convertase TACE (also known as ADAM17) (Healy and Lilic, 2021), are internalized through receptors of the renin–angiotensin system (Yeung et al., 2021).

Additionally, in cells with low ACE2 expression, viral infection can be enhanced by additional host factors, such as the extracellular protease BSG (also known as CD147), the transmembrane receptor NRP1 (Cantuti-Castelvetri et al., 2020), lectins (Lempp et al., 2021), or HAVCR1 phosphatidylserine receptors such TIM-1 (also known as TIMELESS) and AXL (Bohan et al., 2021; Wang et al., 2021b). Studies based on publicly available transcriptome databases revealed that one of the most likely enhancers of direct viral infection in the brain is *NRP1*, which is highly expressed in multiple neuron and glial cell types, including astrocytes and microglia (Cantuti-Castelvetri et al., 2020; Daly et al., 2020; Lonsdale et al., 2013). Recently, the extracellular proteases BSG and DPP4 have also been identified as additional entry receptors for SARS-CoV-2 on astrocytes, providing new detail on the neuropathogenesis of COVID-19 (Andrews et al., 2022; Wang et al., 2020) (Fig. 2).

Regardless of the presence of the virus within the brain, the histological effects of SARS-CoV-2 infection are visible in several brain regions. Postmortem analyses of COVID-19 patients' brain tissue revealed T-cell infiltration and microglial activation, and injured neurons and astrocytes (Kanberg et al., 2020; Schwabenland et al., 2021). Taken together, this extensive body of research confirms that infection with SARS-CoV-2 has complex and wide-ranging effects on the CNS, and helps explain some of the neurological manifestations of acute and long COVID.

## Pathophysiology of long COVID in the CNS

As of 6 October 2021, the WHO has defined long COVID as a condition that begins to develop 3 months after the onset of SARS-CoV-2 infection and involves symptoms that last for at least 2 months and that cannot be explained by an alternative diagnosis (Soriano et al., 2022). Despite the difficulties in adequate diagnosis, long COVID includes a combination of multifactorial symptoms such as fatigue, dyspnea, persistent cough, chest pain, persistent headache, memory and cognitive impairment, muscle aches, loss of smell or taste, depression and/or anxiety (Davis et al., 2023; de Erausquin et al., 2021; Lechien et al., 2020; Vos et al., 2022). Epidemiological studies estimate that 10% of mild acute COVID cases and, depending on the cohort, up to 87.4% of severe acute cases of COVID-19 that required hospitalization experienced at least one long COVID symptom 6 months after discharge (Carfì et al., 2020; Huang et al., 2021). However, in post-critical COVID-19 patients, it becomes difficult to discern whether long COVID symptoms are sequelae of acute infection or arise due to the long hospitalization and/or intensive care interventions (Garrigues et al., 2020; Iadecola et al., 2020).

Some long COVID patients report persistent symptoms 6 months after acute infection, most commonly dyspnea and a diverse array of neurological problems (Box 1, Fig. 1). These reports either describe worsening of symptoms or the appearance of symptoms that were not reported during the acute phase (Fernández-Castañeda et al., 2022; Nalbandian et al., 2021; Nath, 2020). For example, a meta-analysis of ∼10,000 COVID-19 studies revealed the persistence of headache in between 8% and 15% of patients in the first 6 months after SARS-CoV-2 infection (Fernández-de-las-Peñas et al., 2021), and two independent patient cohort studies reported partial taste and smell disorders in 40% of patients (Lechien et al., 2020; Leedman et al., 2021). A similar meta-analysis also revealed that patients of 70 years and above are at higher risk of long COVID, although it is not exclusive to adults (Sudre et al., 2021). Younger patients, including children, can also develop persistent symptoms that are highly similar to those of long COVID in the older population (Blomberg et al., 2021; Buonsenso et al., 2021; Gonzalez-Aumatell et al., 2022; Izquierdo-Pujol et al., 2022; Lewis, 2021; Wang et al., 2021a). Unlike the acute disease, long COVID appears to affect women more often and more severely than men (Huang et al., 2021; Xiong et al., 2021). Along with female sex and older age, low socioeconomic status, smoking, obesity and a large panel of other comorbidities, i.e. chronic obstructive pulmonary disease, prostatic hyperplasia, fibromyalgia, depression or multiple sclerosis, among others, have also been associated with a higher long-term risk of long COVID (Subramanian et al., 2022; Thompson et al., 2022).

**Box 1. Acute SARS-CoV-2 infection in the CNS**

Although coronavirus disease 2019 (COVID-19) is primarily a respiratory disease, patients frequently present with several neurological symptoms such as anosmia, hypogeusia, headaches or cognitive disfunction, as well as with an increased risk of ischemic and microhemorrhagic events during and after infection. However, the pathophysiology of COVID-19 in the brain remains obscure, mainly due to the impossibility of obtaining brain parenchyma biopsies from living patients and to the high cellular and structural complexity and regionalization of the human brain. During acute COVID, the central nervous system (CNS) pathophysiology has been associated with four main predominant events: direct virus-mediated cytopathogenicity, dysregulated immune response, endothelial cell damage and thromboinflammation, and alteration of the renin–angiotensin system (Budhraja et al., 2022; Gupta et al., 2020; Pucci et al., 2021; Vaduganathan et al., 2020). However, there appears to be no consensus within the scientific community about the entry route of severe acute respiratory syndrome coronavirus 2 (SARS-CoV-2) into the CNS. Hypothesized entry routes include the olfactory and trigeminal nerves, cerebrospinal fluid and/or vasculature, matching those of other coronaviruses (Bagheri et al., 2020; Bostancıklıoğlu, 2020; Elmakaty et al., 2022; Luis et al., 2021; Meinhardt et al., 2020; Nader et al., 2021; Tarnawski and Ahluwalia, 2022; Vitale-Cross et al., 2022). Alterations in the blood–brain barrier, owing to pre-existing conditions such as multiple sclerosis and/or associated with the inflammatory response during the acute phase of infection, facilitate the penetration of SARS-CoV-2 into the brain parenchyma (Krasemann et al., 2022; Zhang et al., 2021). It is not yet known to what extent the neurological manifestations of COVID-19 are caused by direct viral replication in the brain, systemic reactions to widespread inflammation, or a combination of the two. The presence of SARS-CoV-2 in postmortem brain samples from COVID-19 patients remains controversial, although an increasing number of COVID-19 autopsies have identified the presence of SARS-CoV-2 particles in the brain parenchyma (Paniz-Mondolfi et al., 2020; Stein et al., 2022).

**Fig. 3.**
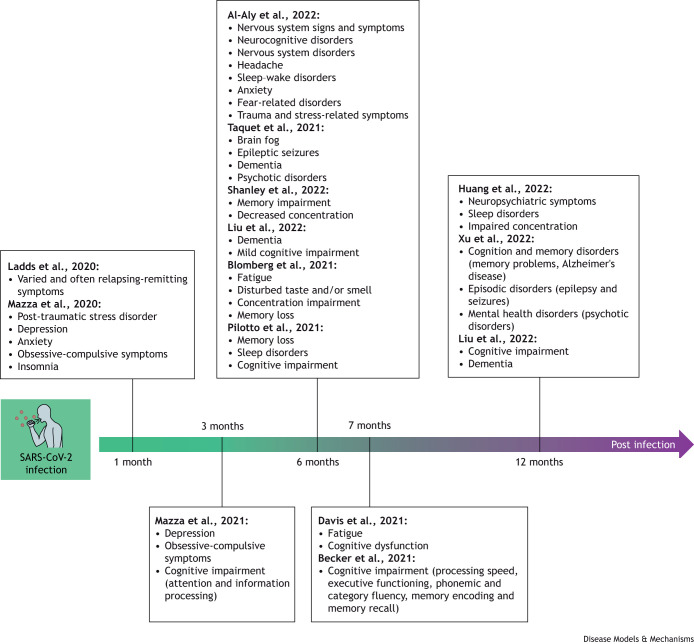
**Neurological symptoms and impairments reported after SARS-CoV-2 infection.** Timeline of the most frequently detected symptoms after SARS-CoV-2 infection.

The most frequently reported manifestations of long COVID are neurological symptoms (hereafter, nLong COVID), which include headache, confusion and ‘brain fog’, mood disorders and dementia (Fig. 3). Researchers have hypothesized that nLong COVID could develop from two main pathological events: persistent brain tissue damage due to acute or relapsed SARS-CoV-2 infection (Box 1) (Buskermolen et al., 2021; Tillett et al., 2021), and/or persistent inflammation of the brain parenchyma upon systemic infection (Bertuccelli et al., 2022; Davidson et al., 2021; Mattioli et al., 2022; Miskowiak et al., 2021; Sneller et al., 2022). A recent study showed that the cytokine CCL11 (eotaxin-1) remains elevated in long COVID patients suffering from brain fog, and high levels of CCL11 have been associated with demyelination of oligodendrocytes and activation of hippocampal microglia (Fernández-Castañeda et al., 2022). However, another study suggests that there is normalization of blood cytokines and other inflammatory biomarkers in nLong COVID (Del Brutto et al., 2022); thus, there is no consensus yet on the persistent inflammation hypothesis. Alternatively, some studies point towards the persistence of cryptic SARS-CoV-2 reservoirs in endothelial cells and/or coactivation of endogenous Epstein-Barr virus (Gold et al., 2021; Stein et al., 2022) as triggers of nLong COVID.

Beyond nLong COVID itself, there is concern about an increased risk of dementia as a longer-term consequence of COVID-19. A number of viruses, including common respiratory viruses, cause post-acute infection syndromes (Choutka et al., 2022), with a significant number of patients developing long-term neurological sequelae, including neurodegenerative multiple sclerosis (Bjornevik et al., 2022; Cermelli and Jacobson, 2000). Human coronaviruses, including SARS-CoV-2, are not an exception, and have neuroinvasive, neurotropic, and potentially direct and indirect neuropathologic capacities (Desforges et al., 2019). Researchers have observed a significant and progressive reduction in COVID-19 patients' gray matter in the parahippocampal gyrus, the entorhinal cortex and the hippocampus after SARS-CoV-2 infection (Di Stadio et al., 2022; Douaud et al., 2022; Griffanti et al., 2021). These regions relate to olfaction and memory function. Accordingly, the risk of being diagnosed with dementia is increased in the 6 months after acute COVID. In the general population, the risk is 0.7% higher, and it rises to 2.7% in patients older than 65 years and to 4.7% in those who have developed encephalopathy during acute infection (Taquet et al., 2021).

Two different scenarios illustrate the potential risk of dementia in the nLong COVID population. First, the most severely hit population by COVID-19 is the age group of 65 years and above (WHO World Health Statistics, 2023), with an increased risk of poor outcome for those with dementia (Numbers et al., 2021). This could be due to a combination of multiple factors, such as the presence of comorbidities and the intrinsic neurocognitive decline associated with aging. Indeed, carrying the Alzheimer's disease risk allele *APOE* ε4 (Kuo et al., 2020; Wang et al., 2021c) or being of an advanced age (González-García et al., 2021) increase the risk of severe COVID-19. Conversely, COVID-19 patients with pre-existing neurological syndromes such as Guillain–Barré syndrome exhibit impairment in amyloid processing along with increased biomarkers of neurodegenerative diseases in their cerebrospinal fluid (CSF), including neuroinflammatory cytokines, glial fibrillary acidic protein and neuronal injury markers (Guasp et al., 2022; Jarius et al., 2022; Kanberg et al., 2020; Pilotto et al., 2021; Ziff et al., 2022; Visser et al., 2022 preprint). Moreover, an increase in soluble ACE2 levels has been detected in patients with cardiovascular and inflammatory conditions, in those with COVID-19 and in the aging population, indicating convergence to the ACE2–renin–angiotensin system (Batlle et al., 2022; Verdecchia et al., 2020). Thus, it remains unknown whether this correlation between COVID-19 and neurodegeneration biomarkers indicates a direct causality or a comorbidity, or, rather, both.

Second, the increased levels of cytokines and chemokines, such as CCL11, upon acute COVID infection are sufficient to induce long-lasting reactivity of white matter microglia in the subcortical and hippocampal regions, compatible with the pathophysiology of cognitive impairment (Etter et al., 2022; Fernández-Castañeda et al., 2022). The reduction in myelinating oligodendrocytes causes loss of myelin and, ultimately, alters cognitive function and neural circuit dynamics (Steadman et al., 2020). Moreover, the presence of antibodies against viral antigens in the CSF of COVID-19 patients suggests a role for autoimmunity in neurological COVID-19 (Song et al., 2021b). Overall, the lack of obvious correlation of long COVID with pre-existing health conditions and with severity of acute COVID underscores the need to introduce novel models to decipher the underlying causes of nLong COVID (Lindan et al., 2021).

## Modeling nLong COVID

If the worst concerns are confirmed and an unprecedented wave of dementias associated with COVID-19 is declared (Arnold, 2020), the expected global socioeconomic impact would easily overpass the current pandemic costs, particularly in the face of the aging population worldwide. Furthermore, other long-term sequelae are an urgent and rising concern, especially to understand the consequences of long COVID in the youngest and to establish early detection and intervention initiatives.

nLong COVID models should recapitulate relevant aspects of the origin and progression of the disease, including the long-lasting effects that may arise months or years after acute COVID. It is essential to understand the pathophysiological mechanisms of nLong COVID from early disease stages. These can originate from virus-driven changes in infected cells/tissues, persistent immunologic aberrations and post-inflammatory damage in response to acute infections, and the expected sequelae of post-critical changes (Nalbandian et al., 2021). Reactivation of SARS-CoV-2 weeks after recovery has been hypothesized after reports of only 9-24% of patients testing negative in consecutive tests (Coppola et al., 2020; Nalbandian et al., 2021; Ren et al., 2021; Ye et al., 2020). One potential cause for this reactivation might be that certain tissues act as viral reservoirs. This possibility should be studied in detail and over longer periods.

Humanized ACE2 mouse models such as the K18 promoter-driven human ACE2-expressing (K18-hACE2) mice and Syrian golden hamsters have been validated for studying the pathology of SARS-CoV-2 infection, vaccines and therapeutics (Frere et al., 2022; Jeong et al., 2022). They have shown the presence of SARS-CoV-2 in neurons, astrocytes and microglia (Kumari et al., 2021; Rhea et al., 2020; Sun et al., 2020). However, humanized animal models are not ideal tools due to the non-physiological expression of ACE2 that could change the tissue tropism and replication of SARS-CoV-2 and, consequently, the validity of the phenotype (Leist et al., 2020; Muñoz-Fontela et al., 2020).

*In vitro* studies of COVID-19, Middle East respiratory syndrome (MERS) and many other zoonoses are restricted by the limited availability of reliable human experimental models that recapitulate the infectivity of the novel viruses in the right human cells. Most two-dimensional (2D) *in vitro* cell models are derived from immortalized cell lines that do not reproduce tissue cytoarchitecture and variability or the expression of relevant proteins for viral infection (Collin et al., 2021). The need for reliable models for human infections has boosted the incorporation of novel technologies, with organoids taking a prominent position in both acute and long COVID research.

## Organoid models for the neurological implications of acute COVID

Organoids are 3D *in vitro* cellular models derived from human stem cells that recapitulate the cytoarchitecture and basic functions of human organs (Clevers, 2016). Organoids can be derived from adult tissue-resident stem cells, embryonic stem cells (ESCs) or induced pluripotent stem cells (iPSCs) reprogrammed from healthy or diseased individuals' somatic cells. As 3D models of human tissues, organoids also have the advantage of mimicking physiological functions. For instance, retinal photoreceptors in human cortical brain organoids respond to light stimulation (Quadrato et al., 2017), brain organoid neurons generate complex oscillatory waves (Trujillo et al., 2019), and airway lung organoids produce mucus (Miller et al., 2019).

In the past decade, infection modeling in organoids has become a reliable tool to understand the impact of Zika virus infection on brain development (Gabriel et al., 2017; Garcez et al., 2016) and of respiratory syncytial virus in the respiratory tract (Sachs et al., 2019). But when the COVID-19 pandemic hit, the potential of organoids to study infectious diseases truly unfolded. Recent studies demonstrate the ability of kidney (Garreta et al., 2022; Jansen et al., 2022a; Monteil et al., 2020), intestine (Giobbe et al., 2021; Lamers et al., 2020; Stanifer et al., 2020; Zhou et al., 2020), colon (Han et al., 2021), liver (Richards et al., 2022; Yang et al., 2020), heart (Mills et al., 2021; Sharma et al., 2020; Wong et al., 2020), lung (Han et al., 2021; Lamers et al., 2021a), brain (Andrews et al., 2022; Mesci et al., 2022; Pellegrini et al., 2020b; Song et al., 2021a) and vascular (Monteil et al., 2020) organoids to model and understand SARS-CoV-2 infection and reveal their potential as a drug-testing platform. For instance, researchers have used iPSC-derived respiratory and colorectal organoids in high-throughput screening of U.S. Food and Drug Administration (FDA)-approved drugs that reduce SARS-CoV-2 replication (Han et al., 2021). Certainly, organoid modeling of acute SARS-CoV-2 infection significantly improved our understanding of its pathobiology in several tissues (Jacob et al., 2020; Lamers et al., 2021b; Samudyata et al., 2022; Tiwari et al., 2021; van der Vaart et al., 2021).

**Fig. 4.**
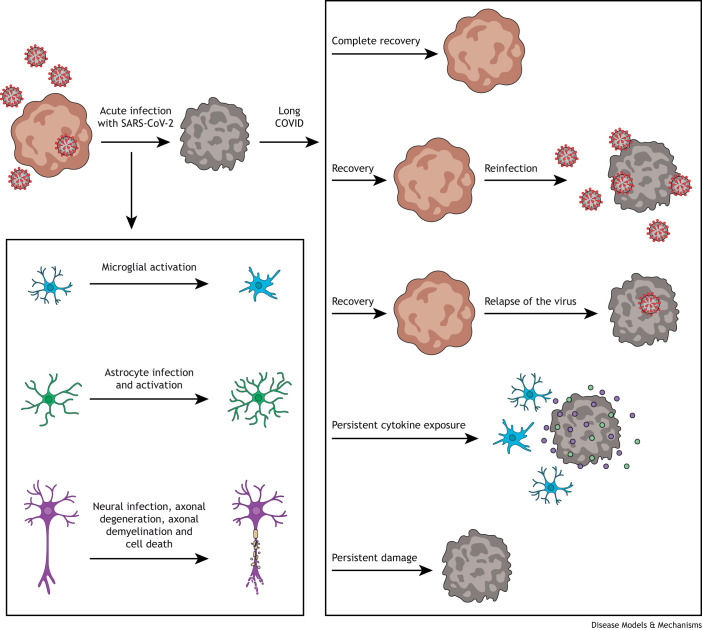
**Brain organoid studies of acute and long COVID.** Cellular changes observed in brain organoids after acute SARS-CoV-2 infection and the potential long-term consequences of viral infection that could be modeled in brain organoids.

Brain organoids have marked a new era, allowing researchers to study the mechanisms that underlie human neurological disorders (Bhattacharya et al., 2022; Nowakowski and Salama, 2022; Xu and Wen, 2021), including those caused by viral infections. Since the first protocol for generating brain organoids was published (Eiraku et al., 2008), many other protocols and uses within the field have been developed. Using these protocols, researchers can generate a wide variety of cell lineage identities, from midbrain, hindbrain and forebrain regions to choroid plexus or retinal structures (Lancaster and Knoblich, 2014a; Pellegrini et al., 2020a; Qian et al., 2018; Velasco et al., 2019). Several modifications have been adopted to overcome some of the well-documented limitations, like the absence of vasculature or poor microglial populations. These include brain-on-a-chip (Bhatia and Ingber, 2014) and co-culture protocols with vascular organoids (Wörsdörfer et al., 2019) or primary microglia (Xu et al., 2021). Although whether human brain cells express ACE2 remains controversial (Fig. 2), several groups, including ours, have detected SARS-CoV-2 replication in infected brain organoids. Some reports indicate that SARS-CoV-2 infects cortical neurons, astrocytes and certain neural progenitor populations (Ramani et al., 2020; Song et al., 2021a; Zhang et al., 2020). The variability of infection rates and phenotypes described in the current literature might be caused by the intrinsic cellular variability derived from brain organoid differentiations, by the use of a wide range of estimated multiplicity of infection (0.00009-10), or by different virus strains (Ramani et al., 2020; Song et al., 2021a; Zhang et al., 2020). Yet, common phenotypes emerge upon analysis of short-term SARS-CoV-2 infection in brain organoids, including increased apoptosis, increased oxidative stress in neurons, myelin disregulation and synapsis loss due to microglial activation (Mesci et al., 2022; Samudyata et al., 2022; Song et al., 2021a). These phenotypes, compatible with progressive neurodegenerative processes, can be devastating to patients due to the limited regenerative capacity of the brain. Additionally, choroid plexus-enriched brain organoids showed viral tropism and infection in the choroid epithelium (Pellegrini et al., 2020b). Interestingly, SARS-CoV-2 infection in brain organoids increased the number of reactive astrocytes adjacent to infected ones, increasing neuronal cell death in the vicinity probably due to a local inflammatory reaction (Andrews et al., 2022) (Fig. 4). The same study suggested that brain organoids expressed ACE2 ectopically due to *in vitro* culture conditions (Fig. 2) (Andrews et al., 2022). Hence, whether human brain cells express ACE2, and how this expression correlates to brain organoids and therefore their validity as SARS-CoV-2 target cells, remains controversial.

## Organoid models for nLong COVID

Upon acute SARS-CoV-2 infection, one can wonder what happens to brain cells adjacent to an infected one – do they acquire features that lead to COVID-19 or even nLong COVID? Neuronal infection increases apoptosis, but how does this affect neuronal circuitry and, consequently, brain function? Brain organoids can be useful models to answer these questions, particularly in the context of nLong COVID pathology. Specifically, they can be used to test whether direct infection with SARS-CoV-2 or the interaction with immune components (by co-culturing organoids with immune cells or by exposing them to soluble immune factors) can trigger phenotypes compatible with nLong COVID. Importantly, two specific features of brain organoid cultures make it possible to explore the long-term effects of acute infection and/or inflammation in a richer variety of settings compared to other models: long-term culture maintenance without passaging and integrative cytoarchitecture with multiple regional identities (Lancaster and Knoblich, 2014b). The organoid system allows the temporal and spatial tracking of the cell that was originally infected, and its surroundings, enabling the observation of any deterioration or regeneration of the cell's physiological features. Moreover, as we have mentioned before, brain organoids can model specific regions that are known to be affected in nLong COVID-19 patients, such as the hippocampus or the prefrontal cortex (Pomeshchik et al., 2020; Ziffra et al., 2021).

Another interesting aspect of brain organoids' differentiation period is that although they retain focal proliferative element groups on 3D neural rosettes for more than a year, some areas reach levels of maturity similar to those of adult human brains in a much shorter timeframe. These mature regions can even develop features of the aged brain, as demonstrated by organoid models of neurodegenerative dementias that developed degenerative phenotypes in 12 weeks (Chen et al., 2021b; Gonzalez et al., 2018). As mentioned above, dementias are among the most frequent COVID-19 comorbidities (Atkins et al., 2020; Barbieri et al., 2022; Martín-Jiménez et al., 2020). Therefore, the accelerated tissue maturation in brain organoids provides a useful tool for modeling the consequence of SARS-CoV-2 exposure *in vitro* and in a compressed timeframe. SARS-CoV-2 infection triggers cellular changes in the brain compatible with the progression of neurodegeneration (Heneka et al., 2020; Yang et al., 2021), which was recapitulated in brain organoid models of acute infection. These organoids showed axonal retraction in infected neurons and dysregulated Tau (also known as MAPT) expression (Ramani et al., 2020). The 3D structure of mature brain organoids is an excellent arena to study changes in the axonal network due to SARS-CoV-2 infection using traditional axonal tracing methods, such as rabies and pseudorabies virus reporters (Ugolini, 2011), or axonal dyes. An unprecedented and yet unexplored issue is the potential effect of multiple infections by different SARS-CoV-2 variants and even different viruses. This emergent matter requires detailed analysis to understand possible viral synergies and enhancement of the pathophysiology, and brain organoids are exquisitely suitable for such studies. Disentangling these synergies is even more important now that multiple variants have evolved and people are being vaccinated and often reinfected (Fig. 4).

Inflammation may play important roles in tissue damage in both severe acute and long COVID (Chen et al., 2021a). Here again, brain organoids are a suitable tool for understanding the short- and long-term cellular and tissue events associated with inflammation (Fig. 4), reducing biases from other infectious processes and/or vaccine-induced inflammation. Furthermore, by treating brain organoids with specific cytokines or patient-derived plasma proteins, researchers can study the role of inflammation over time. An important shortcoming of brain organoids for COVID-19 modeling is the lack of immune cells and of the blood–brain barrier (BBB). These are key in the *in vivo* pathophysiology of acute COVID and nLong COVID (Douaud et al., 2022; Krasemann et al., 2022; Meinhardt et al., 2020; Wenzel et al., 2021). Using mixed cultures of brain organoids, also known as assembloids (Kanton and Pasça, 2022), or co-cultures of neural and non-neural organoids can address this and, additionally, decipher the non-cell-autonomous mechanisms associated with nLong COVID, particularly because the capacity of SARS-CoV-2 to directly infect neurons is still debated. Wang and colleagues infected neural–perivascular assembloids with SARS-CoV-2, and their results suggested that pericytes, which are essential for the maintenance of the BBB, behave as virus replication hubs (Wang et al., 2021d).

Brain organoids, like organoids in general and other model systems, have limitations. We discussed some above. Variability is another key challenge, as even organoids from the same differentiation batch differ due to the stochastic nature of the organoid development process. This is the most criticized aspect of organoid technology. Although partly true, it is arguably a question that arises from comparing organoids with 2D cell cultures. This is still a new and growing field, so the lack of protocol standardization, combined with differences in the origin and genetic background of the source stem cells, might partially explain some of the conflicting results between studies regarding, for example, the capacity of neurons to support replicative infection with SARS-CoV-2 (Bullen et al., 2020; Jacob et al., 2020; Pellegrini et al., 2020b; Ramani et al., 2020; Song et al., 2021a). It is likely that the majority of the variability in the results derives from the experimental conditions of the SARS-CoV-2 infection: the variant and amount of the virus used for the infection, and maturation of the organoids at the time of infection. However, this variability among published studies also highlights the versatility of the model and its capacity to reproduce the biological variability seen in COVID-19 patients. Having said this, brain organoids can be useful for nLong COVID modeling, but should be complemented with other models, particularly animal models such as Syrian hamsters, mice, ferrets and non-human primates, which have shown robustly their use in COVID-19 modeling (Jansen et al., 2022b).

## Conclusions

The high incidence of long COVID, both as a clinical and socioeconomic challenge, is concerning. In particular, the persistent neurological symptoms in these patients are generating concerns about an eventual epidemic of neurodegenerative disorders in the coming years and decades (Tang et al., 2022).

To date, the systemic complexity of long COVID, including its neurological implications, and the risk factors predisposing to the condition remain undetermined. Brain organoids can help researchers dissect the individual neuropathological causes of the disorder in a human- and even a patient-specific manner. Importantly, brain organoids, along with humanized animal models, are the only current technologies that generate prospective results to accelerate the understanding of nLong COVID. When used in conjunction with complementary techniques like epidemiological and clinical studies, these technologies can aid the research community in unraveling the ins and outs of this imminent epidemic. In-depth study of the pathophysiology of a disease as complex and new as nLong COVID requires the combination of multidisciplinary approaches and multiple models and, crucially, reliable natural history data from patients.

As the organoids field matures, these systems are becoming powerful tools for disease modeling. Of course, just like animal models and 2D cultures, organoids have specific limitations and drawbacks, but offer exquisite complementarity to these other models. In addition, brain organoids open possibilities that have remained unexplored until now, such as incorporating a living, patient-derived genetic background with a complex multicellularity. Progress in this field will require the implementation of novel approaches, such as AI-powered algorithms, to analyze them in a deep and comprehensive manner. This will help researchers to gain insights into the intricate mechanisms, risk factors and, hopefully, effective therapies for nLong COVID and other neurological complications resulting from SARS-CoV-2 and other viral infections.
